# Psychedelic Drugs Rediscovered—In Silico Study of Potential Fetal Exposure to Analogues of Psychedelic Drugs During Pregnancy

**DOI:** 10.3390/molecules31020212

**Published:** 2026-01-08

**Authors:** Anna W. Sobańska, Andrzej M. Sobański, Elżbieta Brzezińska

**Affiliations:** 1 Department of Analytical Chemistry, Medical University of Lodz, 90-151 Łódź, Poland; elzbieta.brzezinska@umed.lodz.pl; 2 Faculty of Chemistry, University of Lodz, 91-403 Łódź, Poland; andrzej.sobanski@edu.uni.lodz.pl

**Keywords:** psychedelic drugs, placenta permeability, drug-likeness, QSAR, atomic contributions, synthetic availability

## Abstract

A total of 250 known and novel compounds—ketamine and serotonergic psychedelics or their analogues—designed to target depression, addictions and/or other mental or neurological disorders and developed as “recreational” (illegal) drugs from three chemical families, ergolines, tryptamines and phenylethylamines, were investigated in the context of their ability to cross the human placenta. Using a novel multivariate model involving compounds’ drug-likeness (according to Lipinski’s Ro5), caco-2 membrane permeability, fraction unbound to plasma proteins, steady-state volume of distribution and the total count of heteroatoms (non-carbon atoms with hydrogens included), it was established that the majority of studied compounds are likely to cross the placenta easily, most probably by the passive diffusion mechanism. Atomic contributions of structural elements of studied compounds were investigated using the Morgan fingerprinting algorithm and it was postulated that the fragments promoting transport of compounds across the placenta are carbonyl, hydroxyl, nitro- and phosphoryloxy groups—rigid polycyclic structures, bulky alkyl/aryl groups and halogen atoms restrict the trans-placental passage. All studied compounds are expected to be relatively easily obtained by synthetic routes, which makes them an interesting target for manufacturers of illegal drugs and warrants the need to pursue pharmacological studies of these compounds in silico.

## 1. Introduction

Psychedelics are a subclass of hallucinogenic drugs whose primary activity is to trigger non-ordinary mental states. Serotonin is a neurotransmitter that affects many human behavioural processes [1] and it was established that the major chemotypes of psychedelics—tryptamines, ergolines and phenylalkylamines—are serotonergic psychedelics that share pharmacology as 5-HT2A, 5-HT2B and 5-HT2C receptor agonists [2,3]. According to archeological studies from today’s Iraq, Europe, Mexico and South America, psychedelic mushrooms and plants (including psilocybin, peyote and strands of morning glory) have been used by humans for ritual purposes for at least 10,000 years [4]; religious rituals involving the consumption of psychedelics are still performed in some populations [5]. In the 1960s and 1970s, the recreational use of psychedelic drugs (including LSD synthetized by A. Hoffmann in 1938 and established to have psychoactive properties in 1943) became a part of counter-cultural trends [6]. Interestingly, low doses of psychedelics and other psychoactive compounds are found in some popular, legal dietary ingredients [7].

Serious scientific interest in serotonergic psychedelics began soon after the psychoactive activity of LSD had been discovered [8]. Clinical studies of LSD proved that small doses of this compound cause several types of short-term symptoms in healthy individuals and psychiatric patients, such as the following: (i) visual disturbances; (ii) auditory phenomena; (iii) cutaneous sensations; (iv) taste changes; (v) space perception disturbances; (vi) thought disorders; (vii) mood changes; (viii) sexual excitement; (ix) paranoid features; (x) disturbances of ego-experience; (xi) time disorders; (xii) changes in body image; (xiii) autonomic disturbances and (xiv) drowsiness. At the same time, some positive, long-term effects of low doses of LSD were reported, such as the following: symptoms in patients with schizophrenia or depression improved for periods of up to a few weeks even after a single LSD administration [8]; potential applications of LSD also include the treatment of alcoholism and tobacco addiction [5,9]. Similarly, other classic psychedelic drugs like psylocibin appear to improve emotional empathy and specific memory types such as semantic associations and associative learning [10]. Psylocibin and its analogues seem to be useful in treatments of eating disorders, chronic pain, depression, fear and PTSD [11,12,13], and an interest in “rediscovered” and “repurposed” psychedelic drugs continues to surge [12,14]. Unfortunately, despite the results suggesting that psychedelic drugs may have a positive influence on some psychiatric patients, natural and synthetic compounds associated with psychedelic experience are illegal in many countries [15]. Such restrictions limit the research using these compounds—human studies are replaced by animal (mainly rodent) models, e.g., head-twitch response assay [16] and in silico investigations [17]. Using rat models, it was established that N,N-dimethyltryptamine (DMT) improves mood and reduces anxiety [18] and psychedelics such as LSD or psylocibin have antidepressant effects [19]—with the likely hypothesis that psychedelic tryptamines can induce hallucinogenic and therapeutic effects through activation of the same receptor [20]. Some psychedelics were also found to exert anti-inflammatory effects which can be further investigated in the context of asthma treatment [21]. It is also postulated that some psychedelics are used in studies of neurodegenerative diseases—it was discovered that psylocibin may induce psychotic-like states in lab animals similar to human Parkinson’s psychosis [22].

Several studies focused on explaining the molecular mechanisms responsible for different changes in human perception associated with already known psychedelics [23]. Interactions between psychedelics and 5-HT1A and 5-HT2A receptors were investigated [24] and the effects of structural modifications (both in the indole ring system and in the amine side chain) of 5-methoxytryptamines upon their affinity for 5-HT1A and 5-HT2A receptors were determined. It was discovered that the biological activity of psilocin isomers with the OH group occupying positions C4 to C7 in the indole ring differ due to different ligand-5-HT2AR receptor interactions [25]. Psychedelics’ affinity for plasma proteins was studied with the conclusion that albumin, as the most abundant protein of blood serum, could serve as the biodistributor of psychedelic drugs [26]. Based on mechanistic insights, some promising analogues of natural psychedelics were designed, which are expected to target depression and other psychiatric disorders without the hallucinogenic activity of parent molecules [1,3,24,27,28,29,30,31,32,33,34,35]. In some instances, on the other hand, novel psychoactive substances (e.g., from the chemical families of tryptamines or phenylalkylamines) were developed as new drugs of abuse [36]. The search for novel, psychedelics-related drugs active in the central nervous system prompted the development of some new (bio)synthetic routes [37,38].

Considering an increasing interest in psychedelic drugs and their possible applications in pharmacotherapy of pain, inflammation and mental and neurodegenerative disorders, it is difficult to understand why so little attention has been paid so far to the safety of emerging compounds from this class in the context of pregnancy. A report on pregnancies of a group of 140 women admitting to the use of LSD prior to or during pregnancy suggested that LSD consumption may increase the prevalence of defects in embryos or infants, but the results were inconclusive since some patients from the studied group suffered from infectious diseases or gave a story of ingestion of other illicit drugs during pregnancy [39]. Some other case studies showed that the LSD exposure during pregnancy may lead to ocular anomalies [40] or intrauterine amputations [41]; on the other hand, according to some researchers, no increased offspring fatality or defects among children could be associated with the maternal LSD ingestion [42,43]. The reported results lack systematic approach—properly designed in vivo studies on pregnant females and human embryos exposed to psychedelics are difficult to conduct due to ethical reasons. Some valuable results were obtained from animal (rat, hamster or zebrafish) tests; it was established that there are possible teratogenic effects or incidents of delayed development in infants whose mothers used LSD, hallucinogenic plant-based beverage ayahuasca, mescaline or ecstasy during pregnancy [44,45]. Other risks associated with the exposure of offspring to psychedelic substances during pregnancy and breastfeeding may be a consequence of altered mental states of mothers, leading to disrupted maternal care [46].

Serotonergic psychedelics are promising drugs in the treatment of postpartum depression but there is insufficient evidence regarding the transfer of such compounds to mother’s milk [47].

So far, the pharmacology of psychedelics and their analogues have been studied mainly in the context of their ability to interact with biological targets in the central nervous system [30]. Data on the ability of both legal and illicit analogues of psychedelics from the chemical families of tryptamines, ergolines and phenylethylamines to undergo trans-placental passage are scarce, and in this study, we intended to fill this gap with systematic investigations based on a novel, simple model of the placenta permeability.

## 2. Results and Discussion

### 2.1. Properties of Studied Compounds

Psychoactive compounds investigated in this study belong, with the exception of ketamine, to one of the following three main chemical families: tryptamines (DMT, psylocibin, psilocin), ergolines (e.g., LSD) and phenylethylamines (mescaline and related compounds). The compounds from these families do not differ significantly in the context of the mean values of many pharmacologically relevant physico-chemical properties or biomembrane permeability descriptors (log ***P***, log ***D***, ***caco2***, ***MDCK***, ***PAMPA***, ***nHA*** and ***nHD***) (Supplementary Materials); all of them are drug-like, according to the Lipinski’s Ro5 (***Lipinski*** = 1) [48,49]. There are, however, parameters which differ between the groups, such as the following: (i) ***nRig***—higher in ergolines; (ii) ***PPB***—lower in tryptamines; (iii) ***Fu***—higher in tryptamines; (iv) ***Flex***—higher in phenylethylamines; (v) ***MW***—lower in tryptamines; (vi) ***nRot***—higher in phenylethylamines; (vii) ***nRing***—higher in ergolines; (viii) ***MaxRing***—higher in ergolines; (ix) log***VDss***—lower in ergolines; (x) ***TPSA***—higher in phenylethylamines and (xi) ***nHet***—higher in phenylethylamines (compared to the other two chemical families). Some of these properties, as demonstrated below, are linked to the ability of molecules to cross the placenta, expressed as log ***FM.*** The properties that play a pivotal role in the placenta permeability are ***Lipinski*** (which encodes compounds’ lipophilicity, molecular size and the ability to form H-bonds), ***nHet***, log***VDss***, ***Fu*** and ***caco2***. Despite the similarities between the three chemical families, the key descriptors (with the exception of ***Lipinski***) differ within the families and those differences are sufficient to result in the log ***FM*** values between ca. −0.05 and −0.70.

### 2.2. Psychedelics’ Placenta Permeability

The placenta permeability of compounds is often expressed as log ***FM***—fetus-to-mother blood concentration ratio in the state of equilibrium. The main transport mechanism for small, moderately lipophilic molecules is passive diffusion and the cut-off value between the crossing and non-crossing compounds is usually assumed to be log ***FM*** = −0.52 (to be more precise, Di Fillipo originally suggested that compounds with log ***FM*** > −0.52 cross the placenta easily; compounds with log ***FM*** < −0.82 do not cross the placenta; molecules with log ***FM*** between −0.52 and −0.82 are dubious [50]).

**Figure 1:**
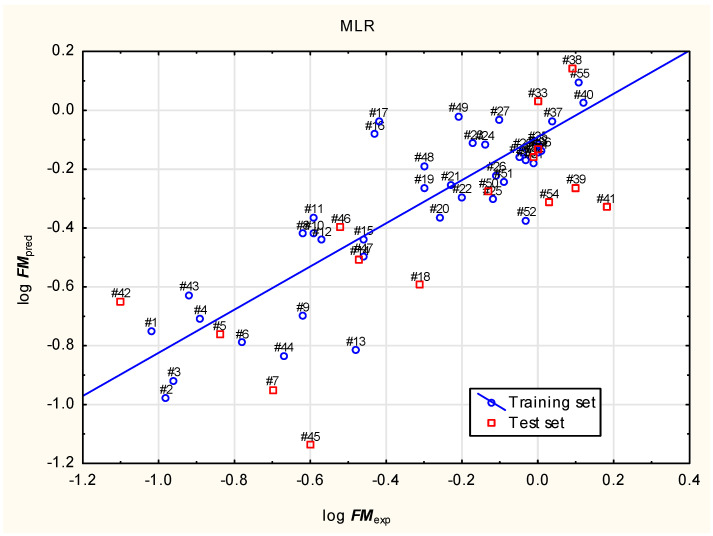
Equation (1)—predicted vs. experimental (observed) log ***FM***.

In our study, the placenta permeability (log ***FM***) of 249 psychedelic compounds and their analogues from three chemical families (tryptamines, ergolines and phenylethylamines; ***57*** to ***305***) and ketamine (***56***) was predicted using a novel Equation (1) based on independent variables selected using the best model method (Figure 1).

log ***FM*** = 0.193 (±0.447) + 0.492 (±0.085) ***Lipinski*** + 0.0410 (±0.0141) ***nHet*** + 0.268 (±0.103) ***caco2*** − 0.172 (±0.080) log***VDss*** + 0.00526 (±0.00132) ***Fu*** (1)

(Training set: n = 40, R^2^ = 0.734, R^2^_adj_. = 0.695, RMSE = 0.184, PRESS = 1.618 and Q^2^ = 0.626; test set: n = 15, R^2^ = 0.580, R^2^_adj_. = 0.518, RMSE = 0.370 and F = 18.7; *p* < 0.001).

The log ***FM*** values calculated according to Equation (1) (Supplementary Materials) imply a high ability of almost all studied compounds to cross the placenta. Some compounds (listed in the Supplementary Materials) have the predicted log ***FM*** values between −0.7 and −0.5—formally around or just below the threshold postulated by Di Filippo (−0.52), but above the value indicating poor placenta permeability (−0.82). However, the lower values of log ***FM*** do not make these particular compounds absolutely safe for a fetus, since the ability of chemicals to reach the fetal circulation is in fact a continuum and even compounds with fetus-to-mother concentration ratio below the threshold are also capable of crossing the placenta to some degree.

**Figure 2:**
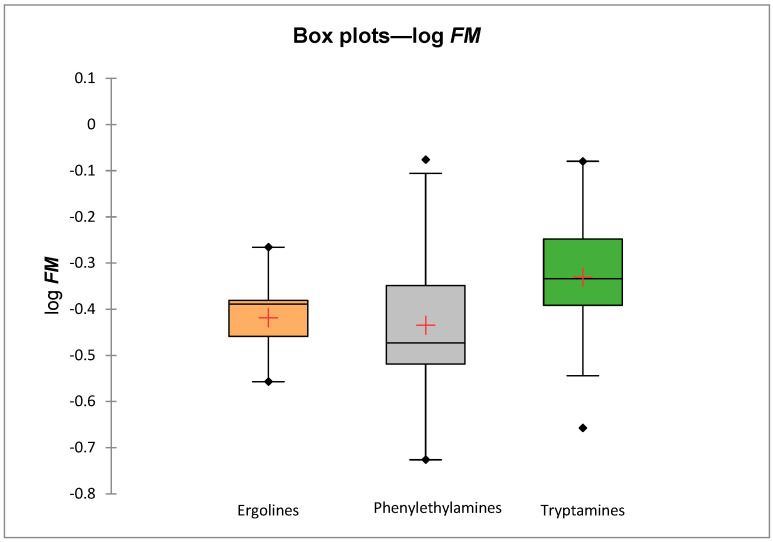
Comparison of predicted mean log ***FM*** values for 3 chemical families.

Predicted log ***FM*** values for three families of psychedelics (tryptamines, ergolines and phenylethylamines) do not differ significantly (Figure 2); they are, on average, slightly higher for tryptamines than for ergolines and phenylethylamines. However, as mentioned above, larger differences in the psychedelics’ ability to cross the placenta were observed within the chemical families.

**Figure 3:**
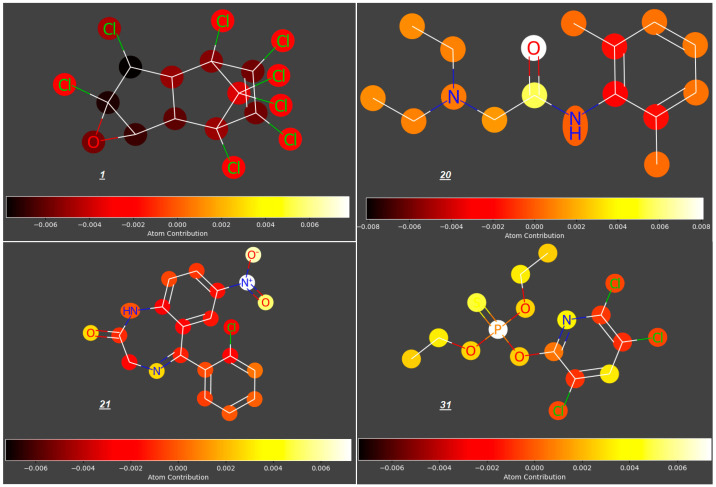

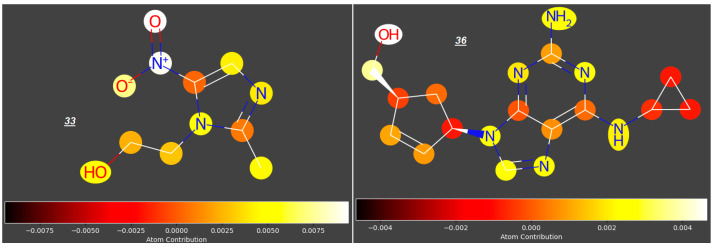
Atomic contributions to low (***1***) and high (***20***, ***21***, ***31***, ***33***, ***36***) placenta permeability.

In this study, we analyzed the contributions of particular atoms to the ability of selected compounds from the studied group to cross the placenta (expressed as log ***FM***). As demonstrated for the reference group of compounds ***1***–***55***, the structural elements promoting transport of compounds across the placenta are carbonyl, hydroxyl, nitro- and phosphoryloxy groups; rigid polycyclic structures and halogen atoms restrict the trans-placental passage (for examples, see Figure 3).

### 2.3. Placenta Permeability of Tryptamines

Psilocybin mushrooms were used ritualistically in pre-Columbian Mexico and their main active constituents, psilocin (***135***) and psilocybin (***131***), were isolated, identified and synthetized in the 1950s [51]. Both compounds became available as “recreational” drugs in the 1960s; however, there is still a gap in knowledge regarding health risks related to the consumption of these substances during pregnancy or breastfeeding. According to our study, their predicted ability to cross the placenta is high, with the log ***FM*** values −0.24 and −0.08 for psilocin and psilocybin, respectively. Other naturally occurring 4-phosphoryloxytryptamines (baeocistin, ***132***; aeruginascin, ***133***; norbaeocistin, ***134***) are also likely to undergo easy trans-placental transport, with log ***FM*** between −0.18 and −0.21—as expected for compounds without bulky elements, halogen atoms or rigid, polycyclic structures and containing phosphoryloxy groups.

Synthetic 4-substituted tryptamines are a serious public health issue, since they became popular (mostly as substances of abuse) relatively recently—mainly in the early 2000s—and there is little information on their pharmacological properties other than the potential to cause psylocibin-like psychedelic effects [32]. As predicted using Equation (1), the 4-HO- and 4-AcO-tryptamines (Figure S1, Supplementary Materials) are likely to cross the placenta easily (4-AcO compounds cross the placenta easier than 4-HO compounds, with log ***FM*** between −0.26 and −0.50 for 4-HO and −0.11 and −0.33 for 4-AcO compounds, respectively). The values of log ***FM*** for the N,N-dialkyltryptamines from the 4-AcO and 4-OH series bearing the same substituents at side-chain N atom are highly correlated (R^2^ = 0.77). The ability of the 4-AcO- and 4-OH-tryptamines to cross the placenta appears to be related to the steric properties of the alkyl groups at the amine nitrogen and it changes in the following order: (i) 4-hydroxytryptamines and 4-acetoxytryptamines with symmetrical alkyl chains DET > DPT > DIPT; (ii) 4-hydroxytryptamines and 4-acetoxytryptamines with asymmetrical alkyl chains MET > EPT > MPT. Similar influence of N,N-alkyl substituents upon activity was reported by Klein et al., who concluded that the crucial factor governing the median effective dose (ED_50_) of N,N-dialkyl tryptamines in mice is the sum of the values of υ (Charton’s parameter υ, based on van der Waals radii) for the two amine substituents [32].

Other possible alteration to the molecular structures of tryptamines is in the pyrrole ring. When this ring is substituted with short alkyl chains or a phenyl group at C2, the ability of compounds to cross the placenta decreases (***137*** vs. ***65***, ***66*** and ***67***, Figure S2, Supplementary Materials), although the differences in log ***FM*** are relatively small (ca. 0.2–0.3 logarithmic unit).

The tryptamines can also be modified at a pyrrole N1 atom. The atomic contributions of substituents at N1 are rather small, resulting in slightly lower log ***FM*** values for compounds ***63***, ***64*** and ***65*** compared to psylocine, ***135*** (ca. −0. 4 vs. −0.24), and a slightly higher log ***FM*** value for compound ***62*** with a 2,2-difluoroethyl group at N1 (Figure S3, Supplementary Materials).

**Figure 4:**
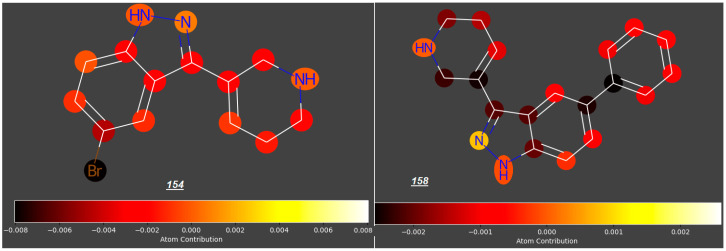
Indazole analogues of tryptamine.

An interesting modification of tryptamines was proposed by Jayakodiarachchi et al. [35], who replaced an indole ring with an indazole ring, thus producing a series of compunds ***145*** to ***149*** and ***151*** to ***158***, two of which (both with a bulky 1,2,3,6-tetrahydropyridin-5-yl substituent at C3 and Br (***154***) or a phenyl group (***158***) at C5) expected log ***FM*** values below −0.6 (Figure 4).

### 2.4. Placenta Permeability of Ergolines

The values of log ***FM*** for ergolines (−0.27 to −0.56) are sufficiently high to suspect that most of them are able to cross the placenta easily. Small difference in log ***FM*** between LSD (***168***) and ***169*** (predicted log ***FM*** values −0.39 and −0.27, respectively) points to a role of a nitrogen N1 atom and the possible impact of a substituent at this atom; however, there is currently no sufficient evidence to confirm this observation (Figure S4, Supplementary Materials). Other reported changes in substituents at N6 (compounds ***168*** and ***171***) and even introducing a Br atom at C2 (compound ***170***) or a strongly electronegative F atom at C13 (compound ***175***) have a limited influence on the predicted ability of ergolines to cross the placenta. One compound (***176***) with a markedly lower log ***FM*** value (−0.54) has a modification at C8 (no carbonyl or hydroxyl oxygen atom in close proximity); even a bulky and rigid (2S,4S)-2,4-dimethylazetidinyl group in ***172*** does not restrict the ability of this compound to cross the placenta sufficiently to warrant safe use during pregnancy. Interestingly, lisuride (***173***), a non-hallucinogenic analogue of LSD approved for the treatment of Parkinson’s disease and high prolactin levels [30], is also expected to cross the placenta easily.

### 2.5. Placenta Permeability of Phenylethylamines

**Figure 5:**
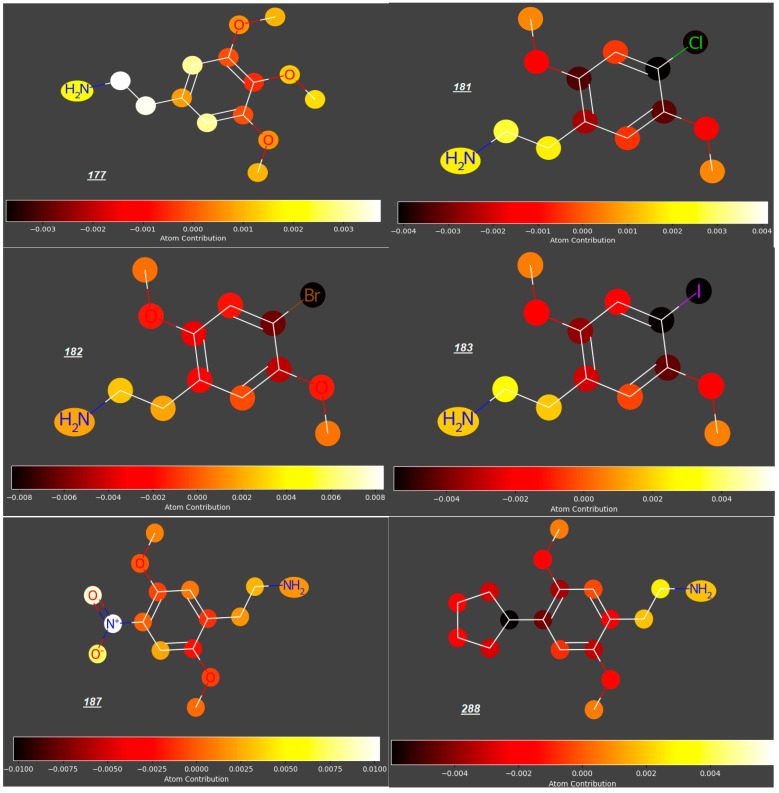
Mescaline (***177***) vs. 2,5-dimethoxy-phenylethylamines with different substituents at C4.

**Figure 6:**
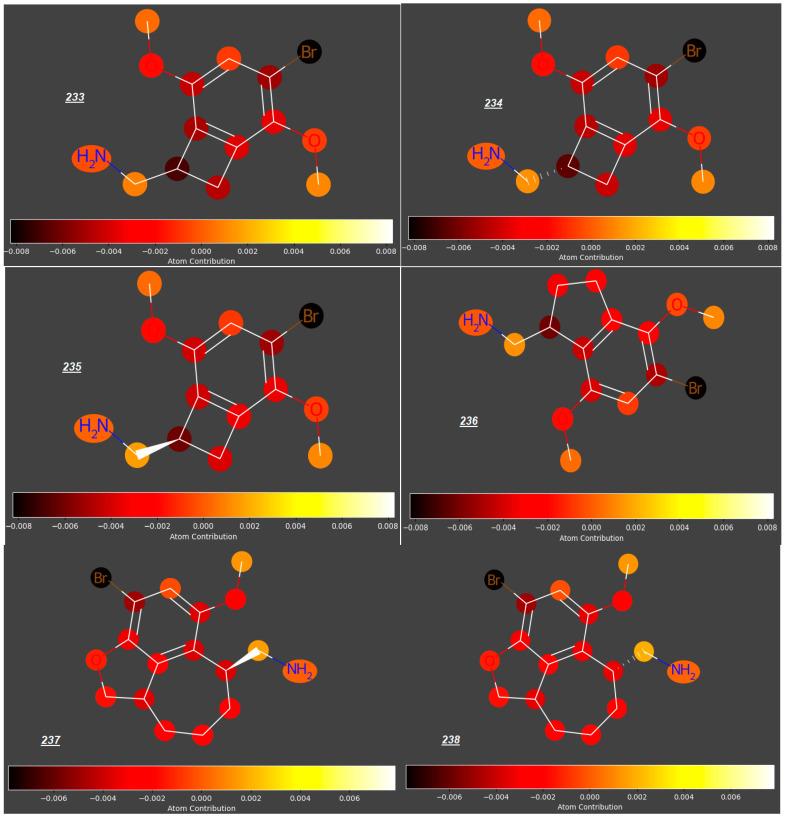
Bi- or tricyclic analogues of mescalines.

Mescaline (***177***), known for ca. 5700 years, identified in the late 1890s and synthesized in 1919 [52], is thought to be relatively safe, with no evidence of cytotoxicity; however, due to its rapid absorption from the gastro-intestinal tract, it may be available in the maternal circulation when administered orally, even despite its relatively fast distribution to kidneys and liver, metabolism and excretion with urine. According to our study, mescaline is a compound with the highest potential to cross the placenta in the whole group of 250 studied compounds (predicted log ***FM*** = −0.09). However, due to even small structural modifications to the phenyl ring or an aminoethyl side chain, other phenylethylamines investigated in this study have significantly lower log ***FM*** values. For some phenylethylamines, the log ***FM*** values between −0.51 and −0.73 are around or even below the threshold quoted by Di Fillipo [50] (−0.52). For example, changing the position of one methoxy group from C3 to C2 and replacing another one (at C4) with halogen atoms or sterically demanding, lipophilic substituents (e.g., a cyclopentyl group) gave compounds ***181*** to ***183*** and ***288*** with much lower log ***FM*** values (−0.54 to −0.63), as opposed to an insignificant reduction in log ***FM*** (to −0.13) for ***187*** with a NO_2_ group occupying C4 position (Figure 5). Compounds ***233***–***238***, whose expected log ***FM*** values are especially low (−0.6 to −0.7), are formally phenylethylamines bearing Br atoms at C4 (Figure 6); however, their aminoethyl side chains are engaged in rigid, cyclic structures condensed with the benzene ring.

**Figure 7:**
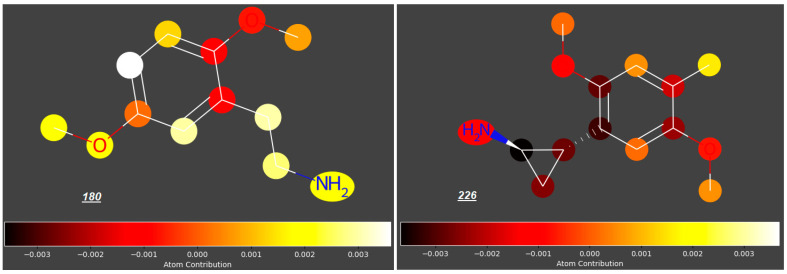

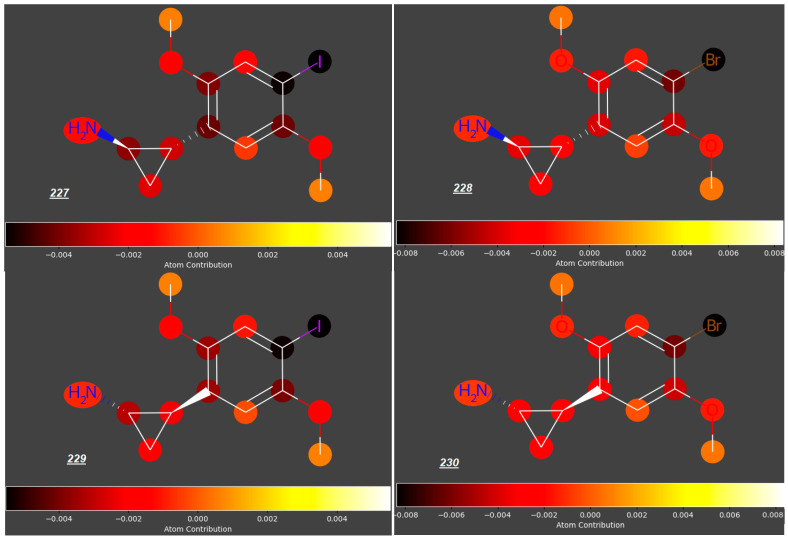
2-(2,5-Dimethoxyphenyl)cyclopropylamines substituted at C4 in the benzene ring.

Significant reduction in the log ***FM*** value could also be observed for mescaline analogues ***226***–***230*** (Figure 7) with an aminoethyl side chain incorporated into a cyclopropyl ring and halide atoms or a methyl group occupying position 4 (log ***FM*** = −0.46 for ***226*** and −0.53 to −0.58 for compounds ***227*** to ***230***, in contrast to log ***FM*** = −0.15 for ***180***). However, considering the low log ***FM*** values for compounds ***181***–***183*** (−0.54 to −0.60), which contain halogen atoms at C4 but no cyclopropyl ring, we concluded that the steric effect of this ring in the side chain is not significant in the context of the placenta permeability compared to the presence of halogen atoms at C4.

In our study, we also investigated other series of compounds, e.g., phenylethylamines with an α-methyl group introduced to the aminoalkyl chain (compounds ***189*** to ***206***). The log ***FM*** values for these compounds are between −0.22 and −0.65, depending on the substituent at C4 in the benzene ring, with bulky and lipophilic hexyl or benzyl groups at C4 causing the most significant reduction in the log ***FM*** values (to −0.65 and −0.60 for compounds ***196*** and ***197***, respectively) and halogen atoms at C4 giving a smaller but marked effect (compounds ***191*** to ***193*** with log ***FM*** = −0.49, −0.54 and −0.41, respectively), as compared to ***194*** with a methyl group at C4 (log ***FM*** = −0.33) (Figure S5, Supplementary Materials).

### 2.6. Synthetic Availability of Psychedelics’ Analogues in the Context of Future Studies

According to our study, compounds ***56*** to ***305*** are likely to be easily absorbed from the gastro-intestinal tract to the maternal circulation (see the Supplementary Materials—GI absorption classified as “High” for all the compounds). We also suspect that compounds ***56*** to ***305*** are able to reach the fetal circulation easily (their transport across the placenta, most probably by the passive diffusion mechanism, may be a little bit more difficult but still possible for some compounds, as discussed above). Unfortunately, many compounds from tryptamine, phenylamine or ergoline families are synthesized as illicit (“recreational”) drugs [36] and this seriously compromises the possibility of studying their pharmacological properties by experimental methods, e.g., due to a limited sample availability. It may also be suspected that there is little interest in such research among the suppliers before the substances hit the illegal drug market and in marking them with proper warnings once they are being sold.

**Figure 8:**
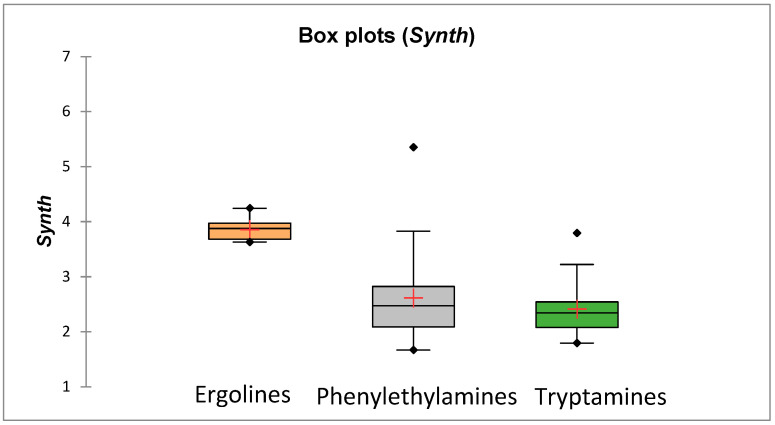
Mean synthetic accessibilities of compounds ***57***–***305***.

In this study, apart from investigations of psychedelics’ drug-likeness and their ability to cross the placenta, we also considered the synthetic availability of studied compounds (predicted based on a combination of fragment contributions and a complexity penalty [53]). It was observed that all the studied compounds should be easy to synthesize (synthesis may be relatively more difficult for ergolines)—there are no compounds in the studied group whose ***Synth*** score would exceed the cut-off = 6 (Supplementary Materials, Table S4 and Figure 8). The possibility of obtaining novel psychedelics’ analogues by synthetic routes (without the need to cultivate and process plants) and the activity of many compounds from this group in the central nervous system make them an attractive target among individuals trying to evade legal controls; also, it should, in our opinion, prompt research on the biological activity of such compounds in the contexts other than their activity in the brain.

### 2.7. Applicability of Equation (1) and Comparison with Other Published Models

The model developed in this study (Equation (1)) is based on compounds with known experimental log ***FM*** values collected by Takaku [54] and later used in other placenta permeability studies [55,56]. The key molecular/membrane permeability descriptors for the reference compounds were compared with those calculated for the psychedelics, with particular focus on the quantitative parameters used in the log ***FM*** model developed herein—***nHet***, ***caco2***, log***VDss*** and ***Fu***. These values obtained for the studied psychedelic compounds largely overlap with those for the reference compounds (Supplementary Materials).

The values of log ***FM*** predicted using Equation (1) were compared with those predicted according to Equations (2) [54] and (3) [55] (calculation results are presented in the Supplementary Materials).

log ***FM*** = −0.00238 ***MW*** +0.00238 ***TopoPSA*** + 0.380 ***Hmax*** + 0.0283 (2)

log ***FM*** = 0.100 − 20.84 ***AATSC1c*** − 0.0132 ***ZMIC1*** (3)

It was established that the Takaku model (Equation (2)) gives results closer to those predicted using Equation (1) than the Wang model and indicates several drugs with relatively low log ***FM***, similarly to (1); the Wang model (Equation (3)) gives relatively high results, with less variability for the studied psychedelics; Wang and Takaku models are not very close to each other. According to Equations (1) to (3), all of the studied compounds are likely to cross the placenta easily (log ***FM*** > −0.52) or belong to the group which Di Filippo defined as “dubious” (log ***FM*** between −0.52 and −0.82).

## 3. Materials and Methods

### 3.1. Compounds

Experimental log ***FM***_exp_ values for compounds ***1*** to ***55***, used to generate quantitative models of placenta permeability described in Section 3, were compiled by Takaku et al. [54] and corroborated by Wang et al. [55]; they are listed in the Supplementary Materials.

Psychedelic compounds and their analogues (***57***–***305***) were taken from Refs. [3,20,24,25,28,30,34].

### 3.2. Calculated Descriptors

Physico-chemical and ADMET properties were calculated using ADMETLab3.0 software based on SMILES strings collected from PubChem. The following physico-chemical descriptors were used in this study: molecular weight (***MW***); van der Waals volume (***Vol***); ***Dense*** = ***MW***/***Vol***; count of hydrogen bond acceptors (***nHA***); count of hydrogen bond donors (***nHD***); number of rotatable bonds (***nRot***); number of rings (***nRing***); number of atoms in the largest ring (***MaxRing***); number of non-carbon atoms including hydrogens (***nHet***); number of rigid bonds (***nRig***); ***Flex*** = ***nRot***/***nRig***; the logarithm of aqueous solubility value (log ***S***); the logarithm of the n-octanol/water partition coefficient (log ***P***); the logarithm of the n-octanol/water distribution coefficients at pH = 7.4 (log ***D***); ***Fsp3*** = number of sp^3^ carbons/total carbon count. Calculated ADMET properties included in the study are the following: (i) membrane permeabilities ***Caco2***, ***MDCK*** and ***PAMPA***; (ii) the volume of distribution at steady state (log***VD*ss**); (iii) plasma protein binding, % (***PPB***); (iv) the fraction unbound in plasma, % (***Fu***). Mordred ***Lipinski*** descriptor was calculated using the OCHEM platform [57]. Binary evaluation of the ability of compounds to be absorbed from the gastro-intestinal tract was assessed using SwissADME platform [58]. Two-dimensional PaDEL descriptors [59] used in predictions according to Takaku [54] and Wang [55] models were calculated using the OCHEM platform [57]. Equation (3) has been modified compared to the original model proposed by Wang to account for non-normalized independent variables.

Calculated molecular descriptors for reference compounds ***1*** to ***55*** and psychedelic compounds/analogues ***56***–***305*** are given in the Supplementary Materials.

### 3.3. Statistical Analysis

Equation (1) was generated in the “best model” mode using XLSTAT 2025.1 from Lumivero, Denver, CO, USA, with 40 compounds randomly assigned to a training set and the remaining 15 compounds used as a test set, with the minimum number of variables = 2 and the maximum number of variables = 5. The best model was selected using the Amemiya’s prediction criterion which penalizes adding predictors more heavily than adjusted R^2^ and thus prevents overfitting [60].

Statistical analysis of physico-chemical and pharmacokinetic properties of compounds was performed using XLSTAT and Statistica v. 13.3 from StatSoft, Kraków, Poland.

### 3.4. Atomic Contribution Analysis

Contribution of molecular fragments to log ***FM*** values was analyzed using ChemMaster Pro v. 1.2 from CrescentSilico. An HQSAR model of log ***FM*** was generated for 55 reference compounds (***1*** to ***55***) and 250 psychedelics (***56*** to ***305***), with molecular fingerprints (circular, 2048 bits, radius = 2, chirality included) as independent variables, with PLS variables pre-selection and 5-fold leave-many-out cross-validation. A total of 75% of compounds were selected at random and used as a training set and the remaining 25% of compounds became a test set. Selected subsets of compounds were then analyzed and the contributions of particular atoms/fragments to log ***FM*** were reviewed manually.

### 3.5. Predicted Synthetic Availability

Synthetic availability of compounds ***56*** to ***305*** was assessed using ***Synth*** score computed with ADMETLab3.0 according to Ertl methodology [53], which returns values between 1 (easy to make) and 10 (very difficult to make). It is assumed that compounds with ***Synth*** ≥ 6 are difficult to synthesize.

## 4. Conclusions

The 250 studied psychoactive compounds are likely to cross the placenta, most probably by passive diffusion—although some phenylethylamines, ergolines and tryptamines have log ***FM*** values around or slightly below the formal cut-off value usually assumed between compounds which cross the placenta easily and those whose trans-placental passage is dubious (log ***FM*** = −0.52). Atomic contributions of structural elements of studied compounds were investigated using the Morgan fingerprinting algorithm and it was observed that the elements promoting the transport of chemicals across the placenta are carbonyl, hydroxyl, nitro- and phosphoryloxy groups; rigid polycyclic structures, bulky alkyl/aryl groups and halogen atoms restrict the trans-placental passage. Based on Equations (1)–(3), there is a high probability of compounds ***56*** to ***305*** being absorbed from the maternal to fetal circulation—no studied psychedelics have log ***FM*** values below the threshold proposed by Di Filippo to identify poor placenta penetrators (log ***FM*** < −0.82).

Our study has some limitations—the predictions of the placenta permeability are made based on a relatively small set of experimental log ***FM*** values compiled from several sources. Additionally, to properly evaluate the risks for offspring, further studies on psychedelics’ possible maternal metabolism and/or routes of excretion from the mother’s body are needed.

## Data Availability

Data are reported in the manuscript.

## Supplementary Materials

The following supporting information can be downloaded at https://www.mdpi.com/article/10.3390/molecules31020212/s1. Figure S1: Atomic contributions of compounds ***122*** and ***124*** to ***130***; Figure S2: Atomic contributions to log ***FM***—5-MeO-DMT derivatives with modifications to the pyrrole ring, at 2 position; Figure S3: Psilocin (***135***) derivatives with modifications to the pyrrole ring, at N atom; Figure S4: Atomic contributions to log ***FM***—ergolines ***168*** to ***176***; Figure S5: Phenylethylamines with an α-methyl group in the aminoalkyl chain, substituted at C4. Table S1: Properties of compounds ***1*** to ***55***; Table S2: Properties of compounds ***56*** to ***305***; Table S3: Relevant properties of reference compounds vs. drugs; Table S4: Predicted synthetic availability of compounds ***56*** to ***305***.
